# VOCs profile can discriminate biological age

**DOI:** 10.18632/aging.202959

**Published:** 2021-04-11

**Authors:** Maria Conte, Giuseppe Conte, Stefano Salvioli

**Affiliations:** 1 Department of Experimental, Diagnostic and Specialty Medicine (DIMES), University of Bologna, Bologna, Italy; 2 Interdepartmental Center, Alma Mater Research Institute on Global Challenges and Climate Change (Alma Climate), University of Bologna, Bologna, Italy; 3 Department of Agriculture, Food and Environment, University of Pisa, Pisa, Italy; 4 Research Center of Nutraceuticals and Food for Health, University of Pisa, Pisa, Italy

**Keywords:** VOCs, volatilome, biological age, diagnostic biomarkers, metabolism

Understanding the biological bases of human aging and longevity has attracted a growing interest over the last decades, as aging is no longer considered as an unavoidable and physiological process, and potential approaches to counteract or delay it are emerging. Moreover, experiments of genetic manipulation on animal models displayed the potential to extend lifespan or prevent many chronic age-related diseases. In this scenario, the need for reliable tools to evaluate the age of the subjects has dramatically emerged, and to this purpose in the last years several studies focused on the identification of new biomarkers that can discriminate people of different age and health status.

Accordingly, our team and others have proposed a number of potential biomarkers, such as epigenetic clocks, serum N-glycans and GDF15, associated to aging or age-related diseases. Moreover, during the last years, our group also studied the “volatilome”, i.e. a set of endogenous Volatile Organic Compounds (VOCs) resulting from body’s metabolism. VOCs are low-weight carbon-based molecules detectable in sweat, exhaled breath, blood, urine and feces, and that, except for blood, are considered non-invasive diagnostic biomarkers. VOCs are involved in different physiological processes and VOCs profile may differ with age, gender, (patho)physiological status, reflecting the metabolic conditions of an individual and represents her/his “odor-fingerprint” [1–3]. A number of studies has already indicated that specific VOCs are associated to diseases, such as cancers, diabetes, malaria, Parkinson’s and Alzheimer’s diseases and can be useful diagnostic tools [2,4,5]. More recently, VOCs belonging to aldehydes and ketones group were found to characterize patients affected by coronavirus disease 2019 (COVID-19) [6]. This observation suggested that infected people could be identified by dogs specifically trained to identify these odorous molecules.

We wondered whether this kind of biomarkers could be useful also to identify people’s age, as suggested by previous studies [7]. We then investigated VOCs profile in healthy aging and longevity in humans by analyzing the VOCs in both urine and feces that better mirror the endogenous metabolism of the organism [8]. The samples derived from volunteers of different age, including centenarians and their offspring that represent a sort of “super-controls” to identify potential VOCs biomarkers of successful aging and longevity. We have reported the existence of specific patterns of urinary and fecal VOCs that can discriminate subjects of different age, from young to centenarians, and, even more interesting, centenarians’ offspring from age-matched controls [8]. Moreover, in order to identify possible VOCs profile influenced by genetics or by environmental factors, such as living place or diet, a comparison between centenarian and her/his offspring or among centenarian, offspring and this latter’s spouse was performed. Interestingly, some VOCs resulted shared between centenarians and their offspring, but not among the trios, indicating the possible existence of a familiar component also for VOCs profile, possibly associated to longevity [8]. Actually, among the different VOCs identified in our study, we found that the fecal VOCs belonging to aldehydes class are less abundant in the group of young with respect to the groups of elders, that generally display a greater susceptibility to inflammation and diseases [3]. This finding is in agreement with literature data indicating that metabolites belonging to aldehydes class are produced during inflammatory processes and are involved in several age-related diseases, such as atherosclerosis, cardiovascular diseases, neurodegenerative diseases and metabolic disorders.

**Figure 1:**
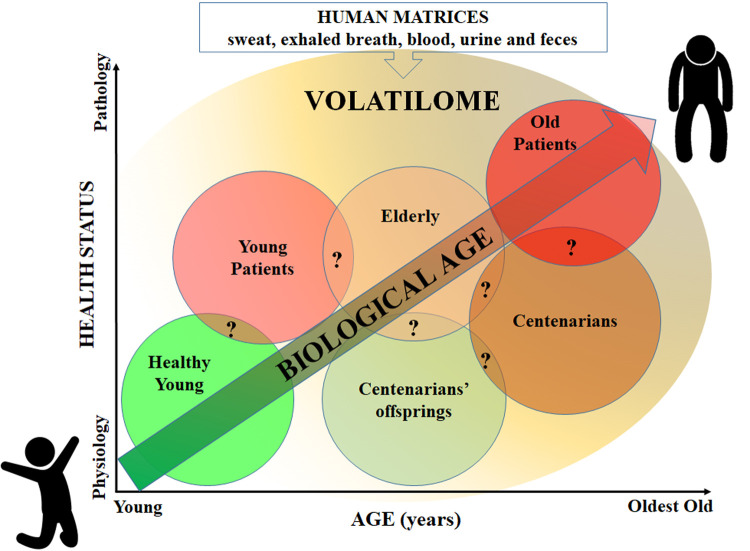
Classification of people groups based on VOCs profile according to the hypothesis that VOCs profile can discriminate age and health status. Question marks indicate possible areas of VOCs overlapping between groups (to be determined).

These observations suggested that the different VOCs patterns may likely reflect changes in metabolic processes associated with age and health status. Therefore, the study of VOCs and “volatilomic” emerged as a valuable approach to identify and/or monitor not only different diseases but also people’s biological age in a non-invasive way. In this perspective, we surmise that groups of people characterized by different age and/or health status could be stratified according to their VOCs profile into a sort of *continuum* from health to disease and from youth to old age (Figure 1). According to this idea, it is assumed that people with a better health status have a lower biological age, and *vice versa*. To identify the boundaries and overlaps of VOCs profiles as well as the true discriminatory power of this approach in order to provide VOCs with a more precise diagnostic value will be the matter of upcoming studies.
